# A comparison of model selection methods for prediction in the presence of multiply imputed data

**DOI:** 10.1002/bimj.201700232

**Published:** 2018-10-23

**Authors:** Le Thi Phuong Thao, Ronald Geskus

**Affiliations:** ^1^ Biostatistics group Oxford University Clinical Research Unit Ho Chi Minh City Vietnam; ^2^ Nuffield Department of Medicine University of Oxford Oxford UK

**Keywords:** lasso, multiply imputed data, prediction, stacked data, variable selection

## Abstract

Many approaches for variable selection with multiply imputed data in the development of a prognostic model have been proposed. However, no method prevails as uniformly best. We conducted a simulation study with a binary outcome and a logistic regression model to compare two classes of variable selection methods in the presence of MI data: (I) Model selection on bootstrap data, using backward elimination based on AIC or lasso, and fit the final model based on the most frequently (e.g. ≥50%) selected variables over all MI and bootstrap data sets; (II) Model selection on original MI data, using lasso. The final model is obtained by (i) averaging estimates of variables that were selected in any MI data set or (ii) in 50% of the MI data; (iii) performing lasso on the stacked MI data, and (iv) as in (iii) but using individual weights as determined by the fraction of missingness. In all lasso models, we used both the optimal penalty and the 1‐se rule. We considered recalibrating models to correct for overshrinkage due to the suboptimal penalty by refitting the linear predictor or all individual variables. We applied the methods on a real dataset of 951 adult patients with tuberculous meningitis to predict mortality within nine months. Overall, applying lasso selection with the 1‐se penalty shows the best performance, both in approach I and II. Stacking MI data is an attractive approach because it does not require choosing a selection threshold when combining results from separate MI data sets

## 1 INTRODUCTION

When creating a prediction model, the aim is to build a clinically useful model with satisfying predictive performance. Variables that are difficult or costly to measure, unreliable, or unavailable at the prediction time are less likely to increase the usability of a prediction model, although their causal relation to the outcome may be strong. In addition, parsimony is a desirable property in predictive modeling. A complex model is often more difficult to understand and communicate. Additionally, it may be overfitted to the data at hand.

Subject matter knowledge and expert opinion should be the most important rationale for selecting a variable in a prediction model (Harrell, 2015). However, this information is not always available, and expert opinion might also introduce bias (Steyerberg, 2009). An alternative option is to perform some form of automated variable selection. For fully observed data, such methods have been extensively studied. The most straightforward, and therefore the most popular variable selection method is backward stepwise selection based on *P*‐value or AIC. The critical shortcomings of this method include instability in selected variables and inflated parameter estimates (Harrell, 2015). Several alternative approaches have been proposed that show more robust performance. Austin and Tu (2004) suggested performing backward elimination on bootstrap samples from the original data. A final model is fitted based on variables with an inclusion frequency over all bootstrap samples above some threshold. In their case study, a threshold of 60% showed the best performance, yet their choice of the considered inclusion frequencies was somewhat arbitrary. Another attractive method is the least absolute shrinkage and selection operator (lasso) selection technique. It combines variable selection and shrinkage via a penalized likelihood technique (Hastie, Tibshirani, & Friedman, 2009). Shrinkage can improve predictive performance, at the cost of some bias in the parameter estimates. Using a simulation study, Van Houwelingen and Sauerbrei (2013) compared the performance of lasso with standard backward elimination followed by some form of shrinkage. The two methods yielded similar performance, with a more parsimonious model obtained by the backward elimination method. The authors considered three fairly strict significant levels of α=0.157,0.05, and 0.01 for backward elimination models, while they only used the optimal penalty in the lasso model. A more parsimonious model is obtained by choosing a stronger penalty, for instance, based on the 1‐se rule (Hastie et al., 2009) or the tolerance rule (Kuhn & Johnson, 2013). However, a stronger penalty may overshrink parameter estimates.

In practice, missing data is commonly encountered, and often handled by multiple imputation (MI). The general idea of the technique is to impute a set of plausible values for missing data based on the distribution of the observed ones. When correctly implemented, MI produces unbiased estimates under missing at random (MAR) mechanisms (Little & Rubin, 2002; White, Royston, & Wood, 2011; van Buuren, 2012). As a rule of thumb, 5‐10 imputations were recommended to yield reliable estimates (Little & Rubin, 2002). Other authors suggested one imputation per percent of missing data (White et al., 2011).

Variable selection in the presence of multiply imputed data needs adaptation. With stepwise backward elimination, the gold standard approach entails fitting the model under consideration to each MI dataset, and combine results via Rubin's rule (Little & Rubin, 2002) to obtain Wald tests for all variables. The least significant variable is removed from the model before moving on to the next step. It is the only method that preserves the type I error; however, it may require intensive computation (Wood, A. M., White, I. R., & Royston, 2008). Numerous approaches have been proposed that are less computationally demanding and can be applied with both backward elimination and shrinkage selection methods. The most straightforward approach is to perform variable selection on each imputed dataset separately and combine results afterwards. The set of selected variables is likely to vary by MI dataset. One option is to fit the final model based on the most frequently selected variables (Wood et al., 2008). The second approach is to combine variable selection with bootstrapping (Heymans, van Buuren, Knol, van Mechelen, & de Vet, 2007; Long & Johnson, 2014). The variable inclusion frequency is calculated over all bootstrap and MI datasets. This method takes into account the uncertainty caused both by missing data and by sampling variability. The third approach is to stack all MI datasets and perform variable selection on that single dataset (Wood et al., 2008). Unlike the previous two approaches, the stacked approach does not lead to different sets of selected variables. When computing *P*‐values in the backward elimination procedure, Wood et al. (2008) additionally proposed a weighting scheme to account for the fraction of missingness per variable, and the repeated occurrence of individuals. Using elastic net as a penalized likelihood variable selection method, Wan, Datta, Conklin, and Kong (2015) adopted the same idea but their proposed weighting scheme is per observation rather than per variable. Chen and Wang (2013) proposed an alternative that applies to lasso selection called MI‐lasso. It is a type of grouped lasso, in which a variable is either not selected at all, or selected in each MI dataset. Contrary to the stacked approach, it may give different coefficient estimates per MI dataset.

A review of current strategies for variable selection in the presence of missing data was given in Zhao and Long (2017). Despite significant contributions, it is unclear that approach performs best and in which circumstances one approach is preferable to the others.

The purpose of this article is to describe and compare strategies for incorporating variable selection with MI data in predictive modeling. We mainly focus on two classes of approaches: (i) lasso or backward elimination in combination with bootstrap sampling; and (ii) lasso selection on the original MI data. The paper is organized as follows. In Section 2, we describe the model selection methods and the performance criteria. In Section 3, we compare the methods using a simulation study with a logistic regression model to predict a binary outcome. In Section 4, we demonstrate the performance of the methods on a real dataset of 951 adult patients with tuberculous meningitis (TBM) to predict mortality within nine months. We discuss our findings in the final section.

## 2 MODEL SELECTION STRATEGIES

We consider a logistic regression model given by:

$$\text{logit}\;\text{P}\left(Y_{i}=1|X_{1i},\ldots ,X_{pi}\right)=\beta_{0}+X_{i}^{T}\beta ,$$

where $Y={\left(Y_{1},Y_{2},\ldots ,Y_{n}\right)}^{T}$ denotes the binary outcome vector of *n* subjects, $X_{i}={\left(X_{1i},X_{2i},\ldots ,X_{pi}\right)}^{T}$ is a set of *p* predictors for subject *i*, $\beta ={\left(\beta_{1},\beta_{2},\ldots ,\beta_{p}\right)}^{T}$ is the vector of *p* regression coefficients.

### 2.1 Lasso models

Lasso coefficient estimates are the solution to the *L*1 optimization problem:

$$\beta_{\mathrm{lasso}}=\text{argmin}\left[-\left\{\frac{1}{n}\sum_{i=1}^{n}Y_{i}\left(\beta_{0}+X_{i}^{T}\beta \right)-\log\left(1+\exp\left(\beta_{0}+X_{i}^{T}\beta \right)\right)\right\}+\lambda {∥\beta ∥}_{1}\right],$$

where ${∥\beta ∥}_{1}=\sum_{j=1}^{p}\left|\beta_{j}\right|$ denotes the *L*1 norm, and λ≥0 is the regularization parameter that controls the amount of shrinkage. Large enough λ will set some coefficients exactly equal to zero, thus lasso performs variable selection. We use a single 10‐fold cross validation for the selection of λ based on the deviance, as implemented in the cv.glmnet function in the R package glmnet (Friedman, Hastie, & Tibshirani, 2010). We consider both the optimal λ and a suboptimal λ based on the 1‐se rule. The former gives the model with the smallest cross‐validated deviance. The latter gives the most regularized model such that the cross‐validated deviance remains within one standard error from the minimum (Hastie et al., 2009). By choosing this suboptimal penalty, we may sacrifice some predictive performance to obtain a more parsimonious model. Due to the stronger penalty, the coefficient estimates are shrunk more towards zero. To correct for the overshrinkage, we consider recalibrating the parameter estimates by refitting the model via unpenalized maximum likelihood, based on the selected predictors or the linear predictor (score) from the lasso procedure. This recalibration is performed on the same dataset that is used to derive the model. Hence, it is different from the classical recalibration methods in validation settings, which are ideally implemented on independent observations. For lasso models with the optimal penalty, we do not perform any type of recalibration as the amount of shrinkage is considered to be optimal.

In more detail, suppose we have a logistic regression model

(1) $$\text{logit}\left(Y\right)=\hat{\beta_{0}}+\hat{\beta_{1}}X_{1}+\hat{\beta_{2}}X_{2}+\cdots +\hat{\beta_{k}}X_{k}$$

with *k* predictors obtained by performing lasso selection on a fully observed data set, where $\hat{\beta }$ is the vector of parameter estimates. The recalibration by score is implemented by fitting a regression model with the score $Z=\hat{\beta_{0}}+\hat{\beta_{1}}X_{1}+\hat{\beta_{2}}X_{2}+\cdots +\hat{\beta_{k}}X_{k}$ as the only covariate, i.e. $\text{logit}\left(Y\right)=\gamma_{0}+\gamma_{1}Z$. The recalibrated coefficients become $\beta_{cal}=\gamma_{1}*\hat{\beta_{i}}$. Hence, the recalibrated model by score retains the relative effects of the lasso regression coefficients in (1). In the more flexible recalibration, we refit a new model based on the *k* selected variables: $\text{logit}\left(Y\right)=\beta_{1new}X_{1}+\beta_{2new}X_{2}+\ldots +\beta_{knew}X_{k}$. The extension to MI data is explained below.

### 2.2 General model selection approaches

We describe the two classes of model selection approaches that we consider in more detail. A summary of all methods is given in Table 1.

i. “Model selection on bootstrap data”: From each of the *m* MI data sets, we repeatedly draw a sample with replacement *B* times. On each bootstrap sample, we perform lasso selection (method BLaF) or backward elimination with AIC as selection criterion (method BBeF). The penalty in the lasso model is identified per bootstrap sample. The proportion of times each variable appears in the mB models is calculated for each method, and a variable is selected if this proportion exceeds x%. We choose x=50. To obtain the final model, we refit the selected predictors to each of the *m* imputed datasets and combine results by averaging coefficient estimates. These approaches are similar to the ones that have been used in Heymans et al. (2007) and Long and Johnson (2014).
ii. “Lasso on original data”: We perform lasso on the original *m* MI data with two different approaches: separate and stacked. In the first one, we apply lasso on each imputed dataset separately and combine estimates by averaging lasso coefficients of predictors that appear in (a) any of the *m* models (method SepAv), or b) in at least x% of the models (method SepAvF). We choose x=50. SepAv is similar to method S1 in Wood et al. (2008), while SepAvF is similar to the method investigated by Lachenbruch (2010) and method S2 in Wood et al. (2008), except that they used backward elimination instead of lasso. For lasso models with the 1‐se penalty, we recalibrate by fitting the score or the selected variables on each MI dataset and averaging the coefficient estimates over all *m* models.

**Table 1:** Summary of considered model selection methods

**Method**	**Description**
FULL	**Full** model with all covariates included
TrueC	Model with all **true** **c**ovariates only (i.e. covariates with nonzero coefficients)
Model selection on bootstrap data	
BBeF	**B**ootstrap resampling. **B**ackward **e**limination (with AIC as the stopping rule) on bootstrap data and fit the final model based on variables with inclusion **f**requency ≥50%
BLaF^a^	**B**ootstrap resampling. **La**sso selection on bootstrap data and fit the final model based on variables with inclusion **f**requency ≥50%
Lasso on original data	
SepAv^a^, ^b^	Lasso selection on each original MI dataset **sep**arately, and **av**erage coefficient estimates
SepAvF^a^, ^b^	Lasso selection on each original MI dataset **sep**arately, and **av**erage coefficient estimates of the variables with inclusion **f**requency ≥50%
Stack^a^, ^b^	Lasso selection on the **stack**ed data
StackW^a^, ^b^	Lasso selection on the **stack**ed data, with a **w**eighting scheme for each observation

a We add letter “o” after the method abbreviation for model obtained by the optimal λ

b We additionally consider recalibration by score and by selected variables

In the stacked approach, we apply lasso on the single stacked data (method Stack). We also investigate the value of adding a weighting scheme for each observation to account for the amount of missingness (method StackW). We employ the same weighting scheme as implemented in method MI‐WENet by Wan et al. (2015). Specifically, each individual *i* is multiplied by a weight $w_{i}$, which is defined as:

$$w_{i}=\frac{1}{m}\left(\frac{\text{Number}\;\text{of}\;\text{non-missing}\;\text{variables}\;\text{of}\;\text{subject}\;i}{p}\right),$$

where *m* is the number of imputations, and *p* is the number of covariates. Hence, subjects with more missing values receive a smaller weight. In the stacked methods, the cross validation procedure for the selection of λ is implemented by individual, i.e. in each fold, a subject is either included *m* times or excluded altogether. By doing so, we account for the variability in the selection of λ incurred by the imputation. For each method that uses the 1‐se penalty, the recalibrated models are obtained by fitting the score or the selected variables on the stacked MI data.

We compare the described methods above with the full model (method FULL) and the model with all true covariates (method TrueC). These two models are obtained by fitting all considered predictors or all true predictors respectively to the stacked MI data.

## 3 SIMULATION STUDY

### 3.1 Simulation design

We consider two data generating mechanisms, differing in the number of covariates *p*, and the correlation structure of the covariates.

#### 3.1.1 Data generating mechanism one: 15 variables (8 noise) with specific correlation structure

We adapt the simulation design from Van Houwelingen and Sauerbrei (2013). Fifteen variables are generated from a multivariate normal distribution with mean 0 and unit variance. We postulate a low overall correlation structure with the corresponding correlation matrix visualized in Supporting Information Figure S1. Specifically, all correlations are 0 except for $\rho_{1,10}=\rho_{2,6}=\rho_{7,14}=\rho_{9,13}=0.5,\rho_{4,8}=-0.7,\rho_{7,8}=0.3,\rho_{11,12}=0.7$. We dichotomize variables $X_{2},X_{4},X_{5},X_{9},X_{10},X_{11},X_{12}$ at 0 to create binary variables. We set 7 covariates, *X*_4_ to *X*_10_, to be true predictors with the following regression coefficients: $\beta_{4}=-0.5,\beta_{5}=\beta_{6}=\beta_{7}=0.5,\beta_{8}=\beta_{9}=1,\beta_{10}=1.5$. The other eight covariates are noise variables with $\beta_{j}=0$. The binary outcome $Y_{i}$ for subject *i* is generated according to: $\text{logit}\;\text{P}\left(Y_{i}=1|X_{1i},\ldots ,X_{15i}\right)=-0.25+X_{i}^{T}\beta$. The empirical prevalence of the outcome is about 60%. The outcome vector *Y* is fully observed, and *X* has missing values. Missing data is generated for variables $X_{2},X_{5},X_{7},X_{8},X_{12}$, and *X*_14_ as specified below.

#### 3.1.2 Data generating mechanism two: 25 variables (18 noise) with first‐order autoregressive (AR(1)) correlation

We generate 25 variables (*X*_1_ to *X*_25_) from a multivariate normal distribution with mean 0 and unit variance, and an AR(1) correlation with ρ=0.5 (the ordering is by variable number) as displayed in the Supporting Information Figure S2. The coefficients and variable types of the first 15 covariates are the same as in the previous data generating mechanism. The extra ten covariates (*X*_16_ to *X*_25_) are continuous variables with coefficients of zero. The prevalence of the outcome is again about 60%. Missing data is additionally generated for variables $X_{16},X_{17},X_{19,}$ and *X*_23_ as specified below.

Denote $R_{ji}$ as the missing indicator for variable $X_{j}$ of subject *i*; $R_{ji}=1$ if $X_{ji}$ is missing and $R_{ji}=0$ otherwise. We only consider scenarios in which the data is MAR (Little & Rubin, 2002). Let *J* denote the set of covariates that contain missing values. The missing data indicator $R_{ji}$ for covariate j∈J of subject *i* is generated following the logistic regression model:

$$\text{logit}\;\text{P}\left(R_{ji}=1|{\left\{X_{ki}\right\}}_{k\notin J},Y_{i}\right)=\alpha_{0}+0.5\sum_{k\notin J}X_{ki}+0.5Y_{i},$$

where α_0_ for each simulation run is estimated by numerically solving the equation:

(2) $$\frac{1}{n}\sum_{i=1}^{n}P\left(R_{ji}=1\right)=miss$$

Here miss is chosen. As the missingness only depends on the observed data, the left hand side of equation (2) is the same for all variables with incomplete data. Thus, on average, the proportion of missing values for any variable $X_{j},j\in J$ equals to miss, and the total proportion of missing values in the data equals to: (The number of variables with missing data*miss)/p.

We impute missing data using multivariable imputation by chained equations (mice) (van Buuren, 2012) as implemented in the R package mice (van Buuren & Groothuis‐Oudshoorn, 2011). Predictive mean matching is used to impute missing continuous variables, and logistic regression is used to impute missing binary variables. The imputation model comprises all predictor variables as well as the binary outcome.

We investigate the performance of the methods described in Section 2 for different values of the proportion of missing values (miss), the sample size, and the number of imputations. Table 2 gives a summary of all the scenarios that we consider in this study. We define the number of events per variable (EPV) as the number of events divided by the number of parameters that is estimated. The number of events is the smaller value when comparing the number of subjects who have the outcome versus the number of subjects who do not (Austin & Steyerberg, 2017). As we fix the probability of events in each data generating scheme, higher EPV corresponds to larger sample size. In a logistic regression model fitted via maximum likelihood, it is recommended that at least 10 EPVs are needed to obtain accurate regression coefficients (Peduzzi, Concato, Kemper, Holford, & Feinstein, 1996). The EPV, the percentage of missing values in the data, and the percentage of complete cases are empirically estimated. For each scenario, 500 datasets are generated.

**Table 2:** Simulation scenarios

	**Data generating mechanism one**	**Data generating mechanism two**
Number of covariates (*p*)	15	25
Proportion of missing values per variable (miss)	0.1, 0.2, 0.3, 0.4, 0.5	0.1, 0.2, 0.3, 0.4, 0.5
The corresponding:		
‐ Percentage (%) of missing values in the data^a^	4, 8, 12, 16, 20	4, 8, 12, 16, 20
‐ Percentage (%) of complete cases^a^	65, 44, 30, 20, 12	65, 47, 34, 24, 16
Sample size n (Events per variable EPV^a^)	200 (5), 400 (10), 600 (15)	200 (3), 400 (7), 600 (10)
Number of imputed datasets (*m*)	10, 20, 30	10, 20, 30
Number of true/noise covariates	7/8	7/18

a Average value

#### 3.1.3 Performance criteria

We assess the predictive performance of the models using Brier score and area under the receiver operating characteristic (ROC) curve (AUC) (Steyerberg et al., 2010). The former measure quantifies how close predictions are to the observed outcome. It combines calibration and discrimination, whereas the latter measure only quantifies the discrimination ability of the model. The evaluation is performed via external validation. A validation dataset is generated from the same data generating mechanism with a sample size of 5000 fully observed observations. We also compare selection performance via the number of selected true and the number of selected noise variables. Better performance is observed with higher value of AUC, larger number of selected true predictors, lower value of Brier score and smaller number of selected noise predictors.

### 3.2 Results

#### 3.2.1 Data generating mechanism one

##### 3.2.1.1 Predictive performance

**Figure 1:**
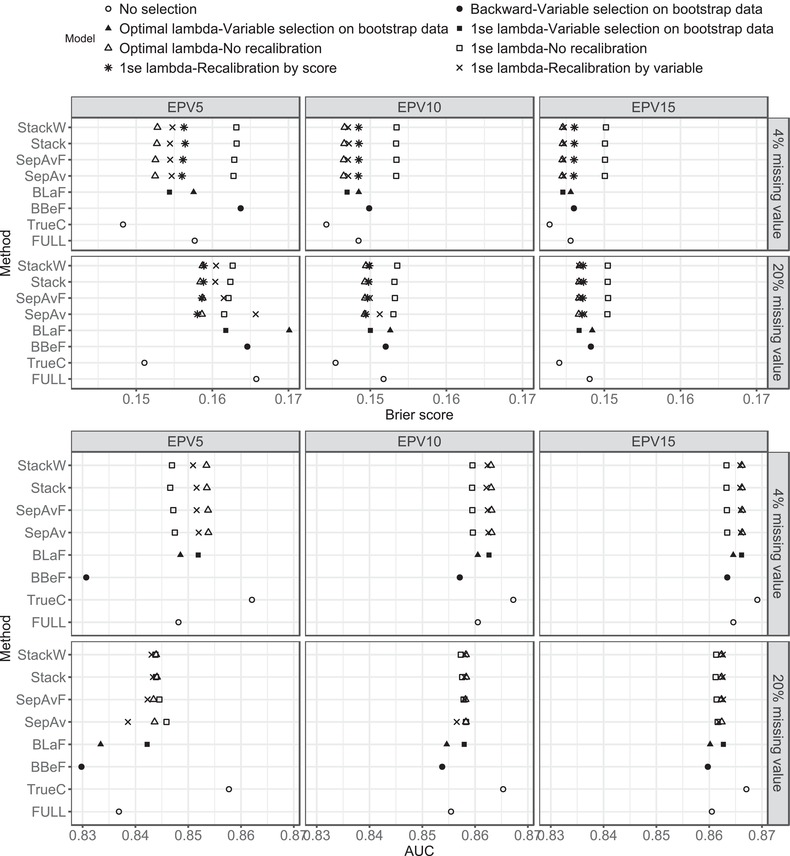
First data generating mechanism (Section 3.1.a): Mean Brier score (top figure) and AUC (bottom figure) over 500 generated datasets for the two most extreme cases of missing values (4 and 20%) with 10 imputed datasets

The mean AUC and Brier score over the 500 generated datasets are presented in Figure 1 for the two most extreme cases of missing values (4% and 20%) with 10 imputed datasets. The results for all scenarios with 10 imputed data together with the ±1 empirical standard deviations are summarised in the Supporting Information Figure S3. Of note, recalibrating a model by score does not change its AUC value because the ordering of the predictions is preserved.

Among model selection on bootstrap data methods, method BLaF with the 1‐se penalty always results in a model with higher AUC and lower Brier score. The difference between the three models (BBeF, BLaF with the two penalties) diminishes with increasing EPV. In the model selection on original data approach, models obtained by the optimal penalty outperform those obtained by the 1‐se penalty without recalibration, except for the AUC value with five EPV and 20% missing. The differences between the two penalty selection strategies are more pronounced in scenarios with 4% missing than with 20% missing data. Hence, the difference in performance seems to increase with a larger amount of information in the data. In data with a low to moderate amount of missing values, recalibrating by selected variables improves model performance. In the scenario with the largest amount of information (4% of missing values and EPV 15), it even yields models with comparable AUC and Brier score to those obtained with the optimal penalty. On the other hand, when the information in the data is low (EPV5 or 10, and 20% missing), recalibrating the models by score gives the better Brier score, and with EPV 5 performance is comparable to the optimal penalty. The most noticeable discrepancy between the two recalibrated models is observed in method SepAv. In fact, after recalibrating by score, the Brier score in SepAv appears to be even lower than when the optimal penalty is chosen in the scenario with 20% missing and five EPV, although the difference is very small (0.158 vs. 0.159).

The Stack and StackW methods yield very similar predictive performance overall. BLaF with the 1‐se penalty provides comparable performance with SepAvF, Stack, and StackW if we recalibrate by the selected variables. The number of imputations has little impact on model performance (see Supporting Information Figure S4). Hence, ten imputed datasets seems adequate in our setting.

Regarding dispersion (see Supporting Information Figure S3), all methods give smaller empirical standard deviation with increasing EPV. The amount of dispersion is similar among methods across all scenarios. The only exception is in data with five EPV and 20% missingness, when method BLaF with the optimal penalty and the recalibration‐by‐selected‐variables version of SepAv show a larger dispersion in their predictive performance results. The widths of the confidence intervals are small in all scenarios, ranging from 0.001 to 0.004 for AUC, and 0.001 to 0.003 for Brier score.

##### 3.2.1.2 Selection performance

**Figure 2:**
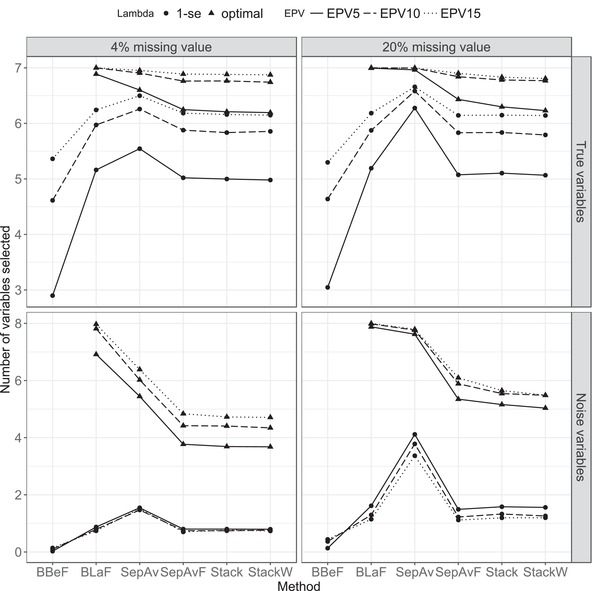
First data generating mechanism (Section 3.1.a): Number of selected variables for the two most extreme cases of missing values (4% and 20%) with 10 imputed datasets

The number of selected true and noise variables are depicted in Figure 2, again for the two most extreme cases of missing values with 10 imputed data sets. As expected, lasso models with the optimal penalty include more variables than those with the 1‐se penalty. It includes most of the true variables, but also a fair amount of noise variables. BLaF with the optimal penalty is the worst method in terms of selection specificity. This method selects nearly all noise variables on average in data with 15 events per variable. If parsimony is the primary concern, this suggests that choosing the optimal penalty is not recommended as it results in a model with very low selection specificity. After BLaF with the optimal penalty, SepAv selects the highest number of both noise and true variables. This is not surprising, as it includes all variables that are selected based on at least one of the imputed datasets. It also selects more noise variables as the number of imputations increases from 10 to 30 (see Supplementary information Figure S5).

Of note, BBeF gives the most parsimonious model in all considered scenarios and selects very few noise variables. However, in data with low EPV, this method on average includes less than half of the number of the true predictors.

Interestingly, models with the optimal penalty include more noise variables as the EPV increases. On the other hand, with increasing EPV, model selection approaches with the 1‐se penalty tend to include nearly the same amount of noise variables in data with 4% missing values, and even fewer in data with 20% missing. The increase in EPV clearly improves the 1‐se penalty models with respect to the number of selected true predictors. On average, less than one true variable is missed if EPV is 15. As the missingness increases from 4 to 20%, all methods select more noise variables. The difference is more pronounced in method SepAv and models with the optimal penalty except for method BLaF as it already selects almost all eight noise variables.

#### 3.2.2 Data generating mechanism two

**Figure 3:**
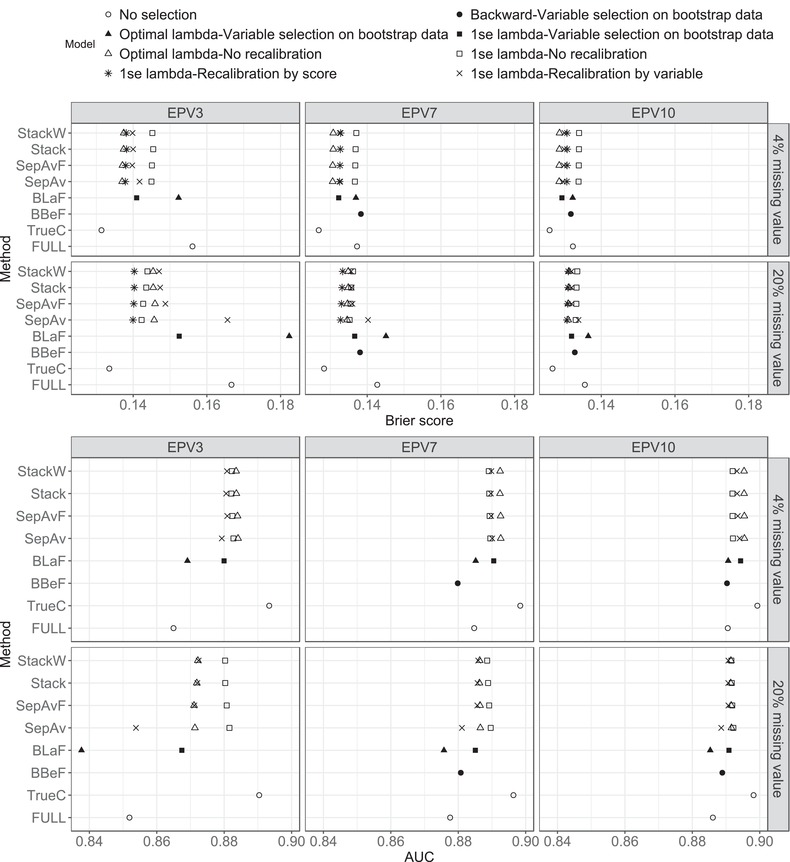
Second data generating mechanism (section 3.1.b): Mean Brier score (top figure) and AUC (bottom figure) over 500 generated datasets for the two most extreme cases of missing values (4% and 20%) with 10 imputed datasets. The performance measures of BBeF in data with EPV of three are not available due to convergence problems in many generated datasets

The predictive and selection performance for the two most extreme cases of missing values with 10 imputed datasets are presented in Figure 3 and Figure S6 in the Supplementary file. With 4% missing, conclusions are very similar to those from the first data generating mechanism. In data with 20% missing, lasso on the original data and recalibrating by score shows the best performance with respect to Brier score in all three EPV scenarios (Figure 3). We observe interesting results in EPV3. Without recalibration, selection models with the 1‐se penalty perform better than the alternatives with the optimal penalty and the BLaF models. Recalibrating by selected variables shows detrimental effect on Brier score and AUC in this data scenario. The dispersion of the predicted performance is presented in the Supporting Information Figure S7. Again, the patterns are very similar to those in the first data generating mechanism. In all methods, the widths of the confidence intervals range from 0.001 to 0.008 for AUC and from 0.001 to 0.005 for Brier score. Just like in the other data generating mechanism, models with the optimal penalty select a substantially larger number of noise variables than models with the 1‐se penalty in all scenarios.

#### 3.2.3 Conclusions from the simulation studies

Using the optimal penalty results in models with better predictive performance compared to the 1‐se rule penalty without correction for over‐shrinkage, unless the amount of information becomes very low. This gain in predictive performance comes with a larger number of selected noise variables. Imposing a stronger penalty, like the 1‐se, is preferable if parsimony is important. Moreover, if we correct for overshrinkage in the 1‐se method, model performance may improve without losing parsimony. In case the EPV is 15, performance approximates that of the optimal penalty if we refit the model based on the selected variables. On the other side of the spectrum, if the amount of information is very low, refitting based on the score can improve the Brier score to values lower than those from the optimal penalty (Note that recalibrating the score has no effect on the AUC). Method BLaF, and the recalibration‐by‐selected‐variables version of SepAvF, Stack, and StackW show similar predictive performance in most of the data scenarios. The recalibration‐by‐selected‐variables version of SepAv achieves similar predictive performance only when the level of missingness is low to moderate. BBeF is the most parsimonious model with comparable predictive performance only in data with high EPV and low percentage of missingness. Note however that we only considered the AIC as the stopping rule for variable selection. Performing backward elimination based on larger *P*‐values will create a less parsimonious model with better predictive performance.

## 4 REAL DATA APPLICATION

### 4.1 Data description

We combine data from three clinical trials in patients with a clinical diagnosis of TBM conducted between 2001 and 2015 by the Oxford University Clinical Research Unit Vietnam (Thwaites et al., 2004, 2011; Heemskerk et al., 2016). A total of 951 HIV‐uninfected patients were included; all were older than 14 years at entry. Our goal is to predict mortality within nine months. Results based on an internally validated Cox proportional hazards regression model can be found elsewhere (Thao et al., 2018). To be consistent with the statistical model used in the simulation study, we use a logistic regression model with nine‐month mortality as outcome. Within those nine months of follow‐up, 219/951 patients died, while 44/951 were lost to follow‐up. As the number of patients lost to follow‐up is less than 5%, excluding them from the analysis will not have a major impact on the results (White et al., 2011).

We applied each of the MI variable selection procedures in Table 1 to the following set of predictors, chosen based on clinical literature and expert opinion: age, weight, sex, treated with dexamethasone, Medical Research Council (MRC) Grade, illness duration at entry, previous tuberculosis (TB), presence of focal neurological signs, body temperature, occurrence of seizures, plasma sodium level, cerebrospinal fluid (CSF) lymphocyte count, CSF protein level, CSF glucose level, ratio of CSF to blood glucose, miliary tuberculosis present on chest radiograph, and cohort. The latter covariate was added in order to account for the changes in treatment, patient management and health care conditions during the time span of the included studies. The first five predictors and cohort are fully observed. The percentage of missing values in the other predictors ranges from 0.1% in temperature to 12.9% in the ratio of CSF to blood glucose. The total percentage of missing values in the data is 2.7%, and the percentage of complete cases is 67.4%. The number of events per variable is 11.7. Ten imputed datasets were generated using the mice package.

We implemented internal validation using the enhanced bootstrap resampling procedure to obtain bias‐corrected values for the performance measures, using 100 bootstrap samples (page 114 Harrell (2015); Musoro, Zwinderman, Puhan, ter Riet, and Geskus (2014)). Specifically, denote the 10 imputed datasets used in the model building process as $Imp_{i},i=1,\ldots ,10$. We bias‐corrected predictive performance measures by subtracting the estimated optimism from the apparent performance, i.e. the performance of the model on the training data. The optimism is calculated as follows: We draw 100 bootstrap samples with replacement from the original dataset with missing data. For each bootstrap sample, we repeat the imputation (denoted as $Imp_{i}^{*},i=1,\ldots ,10$) and variable selection steps as described above. The obtained final model is validated on the 10 bootstrap imputed datasets ($Imp_{i}^{*},i=1,\ldots ,10)$ to quantify the apparent performance, and on the 10 original imputed datasets ($Imp_{i},i=1,\ldots ,10$) to quantify the test performance. The difference between the apparent and test performance is averaged across bootstrap samples and imputed datasets to get an estimate of the overall optimism.

### 4.2 Results

**Figure 4:**
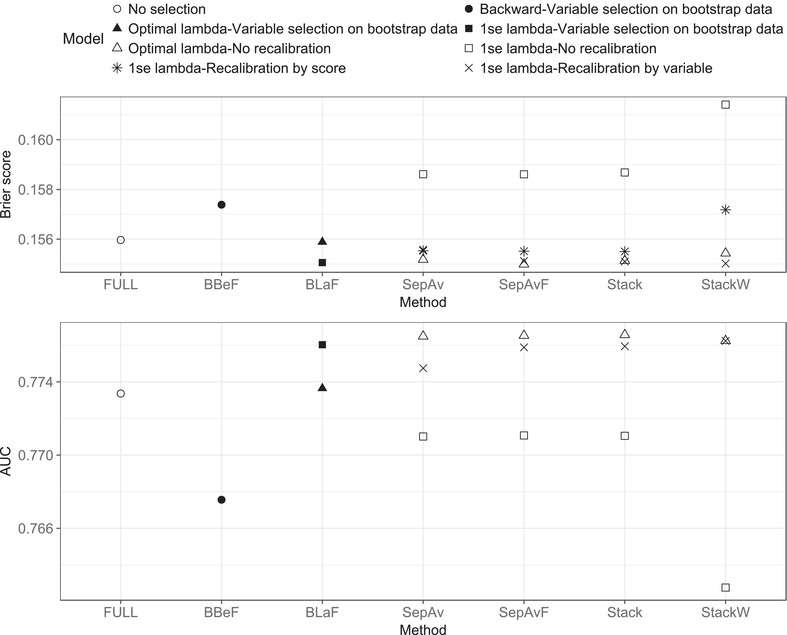
TBM data: Corrected AUC and Brier score via internal validation

Figure 4 presents the bias‐corrected predictive model performance. The results for the variable inclusion frequency by the model selection on bootstrap data methods are presented in the Supporting Information Table S1. In line with the results from the simulations, BBeF yields the most parsimonious model, with only five variables included: Age, MRC Grade, presence of focal neurological signs, CSF lymphocyte count, and cohort. Models obtained by lasso with the 1‐se rule, i.e. BLaF, SepAv, SepAvF, Stack, and StackW, select the same eight predictors (Table 3). After recalibration by selected variables, they give similar bias‐corrected predictive performance measures (AUC ranges from 0.775 to 0.776, Brier score = 0.155) and slightly outperform BBeF (AUC = 0.767, Brier score = 0.157 ) and the full model (AUC = 0.773, Brier score = 0.156 ). The additional three predictors, namely the use of dexamethasone treatment, weight and the history of TB, are easy to measure and do not incur any extra costs in practice. Choosing the optimal penalty, on the other hand, gains minor improvement in the predictive performance at the cost of two to seven extra variables. Two of them, ratio of CSF to blood glucose or CSF protein, require significant extra effort to measure (Table 3).

**Table 3:** Variable selection in the TBM dataset

	**Method**											
**Variables**	BBeF	BLaF	SepAv	SepAvF	Stack	StackW	BLaFo	SepAvo	SepAvFo	Stacko	StackWo	FULL
Age	x	x	x	x	x	x	x	x	x	x	x	x
MRC grade	x	x	x	x	x	x	x	x	x	x	x	x
CSF lymphocyte count	x	x	x	x	x	x	x	x	x	x	x	x
Presence of focal neurological signs	x	x	x	x	x	x	x	x	x	x	x	x
Cohort	x	x	x	x	x	x	x	x	x	x	x	x
Received dexamethasone treatment		x	x	x	x	x	x	x	x	x	x	x
Previous TB		x	x	x	x	x	x	x	x	x	x	x
Weight		x	x	x	x	x	x	x	x	x	x	x
Ratio of CSF to blood glucose							x	x	x	x	x	x
Illness duration at entry							x	x	x	x	x	x
CSF protein							x	x	x	x		x
Occurrence of seizures							x	x				x
Plasma sodium							x	x				x
Miliary tuberculosis present on chest radiograph							x					x
Sex							x					x
CSF glucose												x
Body temperature												x

## 5 DISCUSSION

We described and compared different popular variable selection methods in the presence of multiply imputed data when the aim is to build a prediction model. We assumed the missing data mechanism to be MAR. Results from the simulated data and the real data application were largely similar with respect to the relative predictive performance and number of selected variables. Overall, no method prevails as uniformly best over all scenarios that we considered in the simulation study. Performance depends on the amount of information in the data. Imposing a stronger penalty than the optimal one provides more parsimonious models that may have only slightly inferior and sometimes even better model performance after recalibration. Stacking all MI data is an attractive method. Although using lasso on bootstrap data or on original MI data separately provides just as good performance, it is sensitive to the chosen variable inclusion threshold. Performing backward elimination on bootstrap data with AIC as stopping rule gives the most parsimonious model. We discuss characteristics of each considered method in more detail.

Variable selection on bootstrap data has been widely used in model development. In our simulation, the predictive performance of this approach depends on the selection method and the amount of information in the data. Backward elimination provides a more parsimonious model with inferior predictive performance than lasso. However, performance of backward elimination is likely to depend on the choice for the stopping rule. In our study, we only considered the popular AIC as stopping criterion, which roughly corresponds to a *P*‐value of 0.157. More liberal choices of the significance level, e.g. 0.5 or 0.1, will certainly result in a less parsimonious model with better predictive performance (Steyerberg et al., 2000). In data with a low amount of information, backward elimination may fail to converge (Grogan & Elashoff, 2017). This failure is a consequence of a complete or quasi‐complete separation pattern in the data (Albert & Anderson, 1984). Of note, lasso does not have the same problem due to its regularization properties. On the other hand, in data with a larger number of events per variable and a lower amount of missing values, the performance of backward elimination improves relatively to the other considered methods. Thus, it is a reasonable choice provided that the amount of information is sufficient. Using lasso with the optimal penalty includes almost all considered variables in the final model, leading to poor predictive performance. The reason is that many variables are selected at the 50% threshold, and we need to refit the model with all these selected variables via standard maximum likelihood. A stricter threshold value for the variable inclusion frequency (e.g. 80% or 90%) may result in a more parsimonious model with potentially better performance. With the 1‐se penalty, lasso provides a model with good performance and a reasonable balance between the number of selected true and noise variables.

The approach to build models on bootstrap samples has a couple of drawbacks. First, results are sensitive to the chosen cut‐off value of the variable inclusion frequency. In our TBM data, there was a clear separation between strong and weak predictors for two of the three considered methods (Table S1), which rendered the 50% threshold value sensible. However, in other data settings, this value will not be optimal. Choosing the cut‐off value therefore could be considered as a post hoc decision, i.e. after obtaining the inclusion frequency for all variables. Second, the internal validation for these methods requires double bootstrap to repeat the entire modeling process (i.e. first bootstrap in the model validation step, second bootstrap in the variable selection step). This procedure could be computationally prohibitive (page 71, Harrell (2015)).

“Lasso on original MI data” methods are fairly straightforward to use and require less computation. Lasso selection with the optimal penalty tends to include many noise variables, which makes it less suitable as a variable selection method. Choosing a larger penalty results in a more parsimonious model with fewer noise variables. However, parameters will be shrunk too much that may hamper predictive performance. If we refit the model with the selected variables via unpenalized maximum likelihood, we can improve performance, especially in data with a reasonable number of events per variable (EPV of 10 or more). In data with a low level of information, this refitting may lead to inflated parameter estimates, and potentially a loss in predictive ability. As an alternative, we can recalibrate the model by score, which takes into account the relative predictive strengths of predictors while updating their coefficients. We observed that this type of recalibration improves performance in data with a low level of information.

We can perform lasso selection on each MI dataset separately and combine estimates afterwards. If we simply average all coefficient estimates, we select all variables that appear in at least one imputed dataset. Hence, the number of selected noise variables tends to be large, and increases with the number of imputations. However, we found that in data with a very low level of information, this method tended to give the best predictive performance in terms of the Brier score if coefficients are recalibrated by score. By averaging variables that are selected in at least half of the MI datasets, we attempt to reduce the number of selected noise variables. However, a disadvantage of this method is that it additionally requires choosing a threshold value for the inclusion frequency.

Stacking all MI datasets before analysis provides an interesting alternative that returns a single set of selected predictors and corresponding coefficient estimates. Therefore, the stacked method avoids making any arbitrary decision about the selection threshold (Vergouwe, Royston, Moons, & Altman, 2010). Wan et al. (2015) suggested that it is better to weight individuals by the amount of missing values. In their simulation study, it improved mean squared error compared to stacking without using weights. In our simulations, using such weights tends to result in a slightly more parsimonious model in general. Yet it does not appear to lead to a uniformly better model performance. In fact, after recalibrating the model by selected variables, the two methods give very similar performance in the simulation study as well as in the real data application.

Our study has a few limitations. First, only a couple of methods were considered as we aimed at providing a comparison of pragmatic approaches that are frequently used by most data analysts. Second, we only considered two simulation settings for the proportion of noise variables and three values for the number of events per variables. However, we think that comparable settings are often encountered in medical research. Third, the TBM data has a relatively low amount of missingness. Yet, the data still confirms the relative difference in predictive performance that was found in the simulation study.

In conclusion, we gave an overview of a couple of strategies for variable selection with multiply imputed data in the development of a prognostic model. By means of a simulation study, we provided insights into their strengths and weaknesses. None of the methods that we considered is new, but to our knowledge, this paper is one of the few studies on how to combine results from multiply imputed datasets in a penalized likelihood framework. Also, we do not know of any other study that systematically compares different recalibration methods if a stronger penalty than the optimal one is chosen. With the increasing use of MI to handle missing data, and a growing number of prognostic models being developed, further research in this direction is urgently needed.

## CONFLICT OF INTEREST

The authors have declared no conflict of interest.

## Supporting information

Supplementary Material

Supplementary Information

Supplementary Information

Supplementary Information

Supplementary Information

Supplementary Information

Supplementary Information

Supplementary Information

Supplementary Information

Supplementary Information

Supplementary Information

Supplementary Information

## References

[bimj1946-bib-0001] Albert, A. , & Anderson, J. A. (1984). On the existence of maximum likelihood estimates in logistic regression models. Biometrika, 71, 1–10.

[bimj1946-bib-0002] Austin, P. C. , & Steyerberg, E. W. (2017). Events per variable (EPV) and the relative performance of different strategies for estimating the out‐of‐sample validity of logistic regression models. Statistical Methods in Medical Research, 26, 796–808. 25411322 10.1177/0962280214558972 PMC5394463

[bimj1946-bib-0003] Austin, P. C. , & Tu, J. V. (2004). Bootstrap methods for developing predictive models. The American Statistician, 58, 131–137.

[bimj1946-bib-0004] Chen, Q. , & Wang, S. (2013). Variable selection for multiply‐imputed data with application to dioxin exposure study. Statistics in Medicine, 32, 3646–3659. 23526243 10.1002/sim.5783

[bimj1946-bib-0005] Friedman, J. , Hastie, T. , & Tibshirani, R. (2010). Regularization paths for generalized linear models via coordinate descent. Journal of Statistical Software, 33, 1–22. http://www.jstatsoft.org/v33/i01/. 20808728 PMC2929880

[bimj1946-bib-0006] Grogan, T. R. , & Elashoff, D. A. (2017). A simulation based method for assessing the statistical significance of logistic regression models after common variable selection procedures. Communications in Statistics: Simulation and Computation, 918, 1–14. 10.1080/03610918.2016.1230216 PMC5722241 29225408

[bimj1946-bib-0007] Harrell, F. E. (2015). Regression modeling strategies. Springer Series in Statistics. New York, NY: Springer New York.

[bimj1946-bib-0008] Hastie, T. , Tibshirani, R. , & Friedman, J. (2009). The elements of statistical learning. Springer Series in Statistics. New York, NY: Springer New York.

[bimj1946-bib-0009] Heemskerk, A. D. , Bang, N. D. , Mai, N. T. H. , Chau, T. T. H. , Phu, N. H. , Loc, P. P. , …, Lan, N. N. (2016). Intensified antituberculosis therapy in adults with tuberculous meningitis. New England Journal of Medicine, 374, 124–134. 26760084 10.1056/NEJMoa1507062

[bimj1946-bib-0010] Heymans, M. W. , van Buuren, S. , Knol, D. L. , van Mechelen, W. , & de Vet, H. C. (2007). Variable selection under multiple imputation using the bootstrap in a prognostic study. BMC Medical Research Methodology, 7, 33. 17629912 10.1186/1471-2288-7-33 PMC1945032

[bimj1946-bib-0011] Kuhn, M. , & Johnson, K. (2013). Applied predictive modeling. New York: Springer.

[bimj1946-bib-0012] Lachenbruch, P. A. (2010). Variable selection when missing values are present: a case study. Statistical Methods in Medical Research, 20, 429–444. 20442196 10.1177/0962280209358003

[bimj1946-bib-0013] Little, R. J. A. , & Rubin, D. B. (2002). Statistical analysis with missing data. Hoboken, NJ, USA: John Wiley & Sons, Inc.

[bimj1946-bib-0014] Long, Q. , & Johnson, B. A. (2014). Variable selection in the presence of missing data: Resampling and imputation. Biostatistics, 16, 596–610. 10.1093/biostatistics/kxv003 PMC5156376 25694614

[bimj1946-bib-0015] Musoro, J. Z. , Zwinderman, A. H. , Puhan, M. a. , ter Riet, G. , & Geskus, R. B. (2014). Validation of prediction models based on lasso regression with multiply imputed data. BMC Medical Research Methodology, 14, 116. 25323009 10.1186/1471-2288-14-116 PMC4209042

[bimj1946-bib-0016] Peduzzi, P. , Concato, J. , Kemper, E. , Holford, T. R. , & Feinstein, A. R. (1996). A simulation study of the number of events per variable in logistic regression analysis. Journal of Clinical Epidemiology, 49, 1373–1379. http://www.ncbi.nlm.nih.gov/pubmed/8970487. 8970487 10.1016/s0895-4356(96)00236-3

[bimj1946-bib-0017] Steyerberg, E. W. (2009). Clinical prediction models. Statistics for biology and health. New York, NY: Springer New York.

[bimj1946-bib-0018] Steyerberg, E. W. , Eijkemans, M. J. C. , Harrell, F. E. , & Habbema, J. D. F. (2000). Prognostic modelling with logistic regression analysis: A comparison of selection and estimation methods in small data sets. Statistics in Medicine, 19, 1059–1079. 10790680 10.1002/(sici)1097-0258(20000430)19:8<1059::aid-sim412>3.0.co;2-0

[bimj1946-bib-0019] Steyerberg, E. W. , Vickers, A. J. , Cook, N. R. , Gerds, T. , Gonen, M. , Obuchowski, N. , …, Kattan, M. W. (2010). Assessing the performance of prediction models. Epidemiology, 21, 128–138. 20010215 10.1097/EDE.0b013e3181c30fb2 PMC3575184

[bimj1946-bib-0020] Thao, L. T. P. , Heemskerk, A. D. , Geskus, R. B. , Mai, N. T. H. , Ha, D. T. M. , Chau, T. T. H. , …, Wolbers, M. (2018). Prognostic models for 9‐month mortality in tuberculous meningitis. Clinical Infectious Diseases, 66, 523–532. 29029055 10.1093/cid/cix849 PMC5850565

[bimj1946-bib-0021] Thwaites, G. E. , Bang, N. D. , Dung, N. H. T. , Quy, H. T. , Oanh, D. T. T. , Thoa, N. T. C. , Hien, N. Q. , et al. (2004). Dexamethasone for the treatment of tuberculous meningitis in adolescents and adults. The New England Journal of Medicine, 351, 1741–1751. 15496623 10.1056/NEJMoa040573

[bimj1946-bib-0022] Thwaites, G. E. , Bhavnani, S. M. , Chau, T. T. H. , Hammel, J. P. , Torok, M. E. , Van Wart, S. A. , …, Ambrose, P. G. (2011). Randomized pharmacokinetic and pharmacodynamic comparison of fluoroquinolones for tuberculous meningitis. Antimicrobial Agents and Chemotherapy, 55, 3244–3253. 21502621 10.1128/AAC.00064-11 PMC3122453

[bimj1946-bib-0023] van Buuren, S. (2012). Flexible imputation of missing data. Boca Raton, FL: Chapman and Hall/CRC.

[bimj1946-bib-0024] van Buuren, S. , & Groothuis‐Oudshoorn, K. (2011). Mice: multivariate imputation by chained equations in R. Journal of Statistical Software, 45, 1–67. http://www.jstatsoft.org/v45/i03/.

[bimj1946-bib-0025] Van Houwelingen, H. C. , & Sauerbrei, W. (2013). Cross‐validation, shrinkage and variable selection in linear regression revisited. Open Journal of Statistics, 3, 79–102.

[bimj1946-bib-0026] Vergouwe, Y. , Royston, P. , Moons, K. G. M. , & Altman, D. G. (2010). Development and validation of a prediction model with missing predictor data: a practical approach. Journal of Clinical Epidemiology, 63, 205–214. 19596181 10.1016/j.jclinepi.2009.03.017

[bimj1946-bib-0027] Wan, Y. , Datta, S. , Conklin, D. J. , & Kong, M. (2015). Variable selection models based on multiple imputation with an application for predicting median effective dose and maximum effect. Journal of Statistical Computation and Simulation, 85, 1902–1916. 26412909 10.1080/00949655.2014.907801 PMC4583148

[bimj1946-bib-0028] White, I. R. , Royston, P. , & Wood, A. M. (2011). Multiple imputation using chained equations: Issues and guidance for practice. Statistics in Medicine, 30, 377–399. 21225900 10.1002/sim.4067

[bimj1946-bib-0029] Wood, A. M. , White, I. R. , & Royston, P. (2008). How should variable selection be performed with multiply imputed data? Statistics in Medicine, 27, 3227–3246. 18203127 10.1002/sim.3177

[bimj1946-bib-0030] Zhao, Y. , & Long, Q. (2017). Variable selection in the presence of missing data: Imputation‐based methods. Wiley Interdisciplinary Reviews: Computational Statistics, 9, e1402 29085552 10.1002/wics.1402 PMC5659333

