# Supplementary material for: A comparison of model selection methods for prediction in the presence of multiply imputed data
Source: Biom J. 2018 Oct 23;61(2):343–56. doi: 10.1002/bimj.201700232 (PMC6492211; doi:10.1002/bimj.201700232)
Supplement: Supplementary file 1 — Supplementary Material [file BIMJ-61-343-s001.zip › Code-submission/Readme.pdf]

README

Thao Le

September 13, 2018

Model selection methods for prediction

This repository contains the code for the paper entitled: *A comparison of model selection methods for prediction in the presence of multiply imputed data* by Le Thi Phuong Thao and Ronald Geskus.

The author mainly responsible for writing the code and whom readers should approach with questions or bug reports is Le Thi Phuong Thao.

The folder `case_study` has the following contents:

- The folder `data` contains the following items:
  - `pooled_data.Rdata`: The TBM data
  - `Realdata_result.Rdata`: R work-space with the results for the real data application (Section 4 of the paper)
- `Funs-realdata.R`: supporting functions to perform variable selection on the real data, and to calculate AUC and Brier score for each multiply imputed data set.
- `Run_realdata_biometrical.R`: an R script file that loads the TBM data set and the supporting functions, performs variable selection on the TBM data and calculates the apparent and the optimism estimates of the model predictive performance.
- `Generating-plot-realdata.R`: an R script file that computes the bias-corrected model performance, and generates Figure 4, Table 3 and Table S1 in the paper. As the execution of `Run_realdata_biometrical.R` is normally quite long, user can use the intermediate results saved in the `data` folder to reproduce the results.

The folder `simulation` has the following contents:

- The folder `intermediate-result` contains the corresponding R work-spaces with the simulation results. Each work-space contains AUC value, Brier score, and coefficients of selected variables over 500 generated data sets for all considered methods for a particular data generating scenario. For example, the work-space `R15_200_10_01.Rdata` stores the simulation results of the following scenario:
  - Number of covariates (p): 15
  - Sample size (n): 200 subjects
  - Number of imputations (m):10
  - Percent of missing value per variable (miss): 0.1.
- `1_gendata.R`: supporting functions to
  - Generate data for the two data generating mechanisms as described in section 3.1 of the paper.
  - Perform multiple imputation for the simulated data
- `2_Funs.R`: supporting functions to perform variable selection on the simulated data, and to calculate AUC and Brier score
- The R script files `3_R15_200_10_01.R`, `3_R15_200_10_02.R`, `3_R15_200_10_03.R`, `3_R15_200_10_04.R`, `3_R15_200_10_05.R`, `3_R15_200_20_05.R`, `3_R15_200_30_05.R`, `3_R15_400_10_01.R`, `3_R15_400_10_02.R`, `3_R15_400_10_03.R`, `3_R15_400_10_04.R`, `3_R15_400_10_05.R`, `3_R15_600_10_01.R`, `3_R15_600_10_02.R`, `3_R15_600_10_03.R`, `3_R15_600_10_04.R`, `3_R15_600_10_05.R`, `3_R15_600_20_01.R`, `3_R15_600_30_01.R`, `3_R25_200_10_01.R`, `3_R25_200_10_05.R`, `3_R25_200_20_05.R`, `3_R25_200_30_05.R`, `3_R25_400_10_01.R`, `3_R25_400_10_05.R`, `3_R25_600_10_01.R`, `3_R25_600_10_05.R`, `3_R25_600_20_01.R`, and `3_R25_600_30_01.R` that load the supporting functions and run the simulation for considered scenarios in the paper. For example, R script file `3_R15_200_10_01.R` corresponds to the following scenario:
  - Number of covariates (p): 15
  - Sample size (n): 200 subjects

- Number of imputations (m):10
  - Percent of missing value per variable (miss): 0.1.
- `4-Generating-plots-submit.R`: an R script file that produces the figures with the simulation results: Figure 1-3, Figure S1-S7. As the execution of simulation is normally quite long, user can use the intermediate results saved in the `intermediate-result` folder to generate the results.

The results have been produced in R under the following specification. Of note, for the code to execute `glmnet` version `2.0-13` or older is required.

```
R version 3.4.3 (2017-11-30)
Platform: x86_64-w64-mingw32/x64 (64-bit)
Running under: Windows 7 x64 (build 7601) Service Pack 1

Matrix products: default

locale:
[1] LC_COLLATE=English_United States.1252  LC_CTYPE=English_United States.1252
[3] LC_MONETARY=English_United States.1252 LC_NUMERIC=C
[5] LC_TIME=English_United States.1252

attached base packages:
[1] parallel  grid      stats     graphics  grDevices utils     datasets  methods
[9] base

other attached packages:
 [1] doParallel_1.0.11  iterators_1.0.8    doRNG_1.6.6        rngtools_1.2.4
 [5] pkgmaker_0.22      registry_0.5       DescTools_0.99.23  glmnet_2.0-13
 [9] foreach_1.4.3      Matrix_1.2-12      rms_5.1-1          SparseM_1.77
[13] Hmisc_4.1-1        Formula_1.2-2      survival_2.41-3    mice_2.46.0
[17] lattice_0.20-35    RColorBrewer_1.1-2 corrplot_0.84      knitr_1.17
[21] gridExtra_2.3      forcats_0.2.0      stringr_1.2.0      dplyr_0.7.4
[25] purrr_0.2.4        readr_1.1.1        tidyr_0.7.2        tibble_1.3.4
[29] ggplot2_2.2.1      tidyverse_1.2.1    rlist_0.4.6.1      mvtnorm_1.0-6

loaded via a namespace (and not attached):
 [1] nlme_3.1-131        lubridate_1.7.1     httr_1.3.1          rprojroot_1.2
 [5] tools_3.4.3         backports_1.1.1     R6_2.2.2            rpart_4.1-11
 [9] lazyeval_0.2.1      colorspace_1.3-2    nnet_7.3-12         mnormt_1.5-5
[13] compiler_3.4.3      cli_1.0.0           rvest_0.3.2         quantreg_5.34
[17] htmlTable_1.11.0    expm_0.999-2        xml2_1.1.1          sandwich_2.4-0
[21] scales_0.5.0        checkmate_1.8.5     polspline_1.1.12    psych_1.7.8
[25] digest_0.6.12       foreign_0.8-69      rmarkdown_1.8       base64enc_0.1-3
[29] pkgconfig_2.0.1     htmltools_0.3.6     manipulate_1.0.1    htmlwidgets_0.9
[33] rlang_0.1.4         readxl_1.0.0        rstudioapi_0.7      bindr_0.1
[37] zoo_1.8-0           jsonlite_1.5        acepack_1.4.1       magrittr_1.5
[41] Rcpp_0.12.14        munsell_0.4.3       stringi_1.1.6       multcomp_1.4-8
[45] yaml_2.1.15         MASS_7.3-47         plyr_1.8.4          crayon_1.3.4
[49] haven_1.1.0         splines_3.4.3       hms_0.4.0           boot_1.3-20
[53] reshape2_1.4.2      codetools_0.2-15    glue_1.2.0          evaluate_0.10.1
[57] latticeExtra_0.6-28 data.table_1.10.4-3 modelr_0.1.1        MatrixModels_0.4-1
[61] cellranger_1.1.0    gtable_0.2.0        assertthat_0.2.0    xtable_1.8-2
[65] broom_0.4.3         bindrcpp_0.2        cluster_2.0.6       TH.data_1.0-8
```
