# Supplementary material for: A comparison of model selection methods for prediction in the presence of multiply imputed data
Source: Biom J. 2018 Oct 23;61(2):343–56. doi: 10.1002/bimj.201700232 (PMC6492211; doi:10.1002/bimj.201700232)
Supplement: Supplementary file 2 — Supplementary Information [file BIMJ-61-343-s002.pdf]

Supporting information for the paper: ‘A comparison of model selection methods for prediction in the presence of multiply imputed data’

Le Thi Phuong Thao[1] and Ronald Geskus[1,2]

[1]Oxford University Clinical Research Unit, Ho Chi Minh City, Vietnam
[2]Nuffield Department of Medicine, University of Oxford, United Kingdom

September 24, 2018

Table S1: Variable inclusion frequency (%) for the model selection on bootstrap data methods in the TBM data

**Variables**	**Method**		
	BBeF	BLaF	BLaFo
Age	100.0	99.9	100.0
MRC grade	99.0	100.0	100.0
CSF lymphocyte count	97.4	100.0	100.0
Presence of focal neurological signs	59.8	93.0	99.9
Cohort	66.8	84.7	98.9
Weight	40.2	70.6	95.6
Previous TB	25.7	68.3	92.1
Received dexamethasone treatment	24.6	61.6	87.0
Illness duration at entry	12.8	36.9	82.7
Ratio of CSF to blood glucose	14.9	22.7	78.1
CSF protein	5.5	6.9	61.5
Plasma sodium	2.1	5.0	61.8
Occurrence of seizures	1.3	3.1	62.6
Miliary tuberculosis present on chest radiograph	2.2	3.7	55.1
Sex	1.8	2.1	53.3
Body temperature	2.6	2.0	48.8
CSF glucose	4.4	1.9	44.2

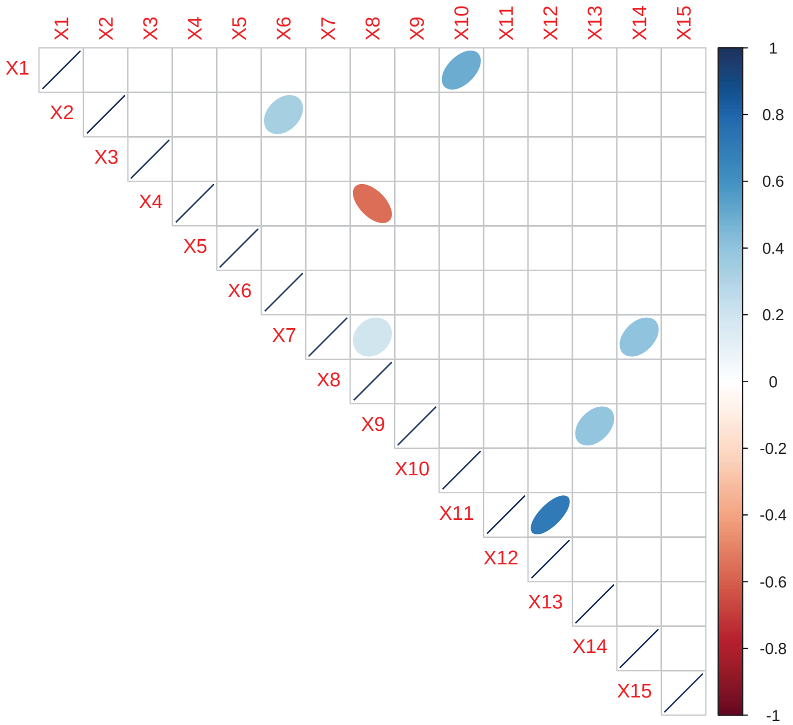

Figure S1: First data generating mechanism: Correlation matrix of the covariates

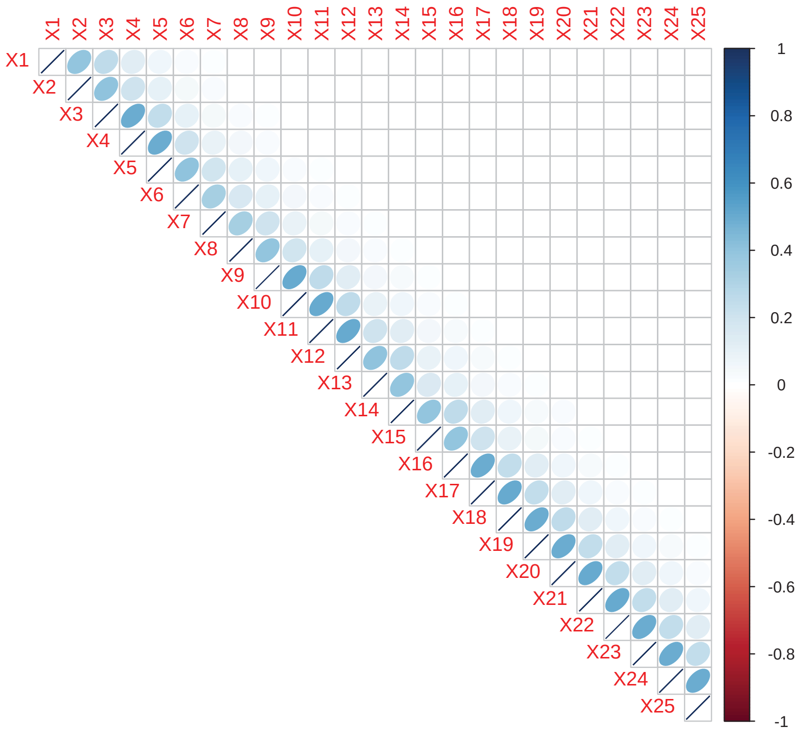

Figure S2: Second data generating mechanism: Correlation matrix of the covariates

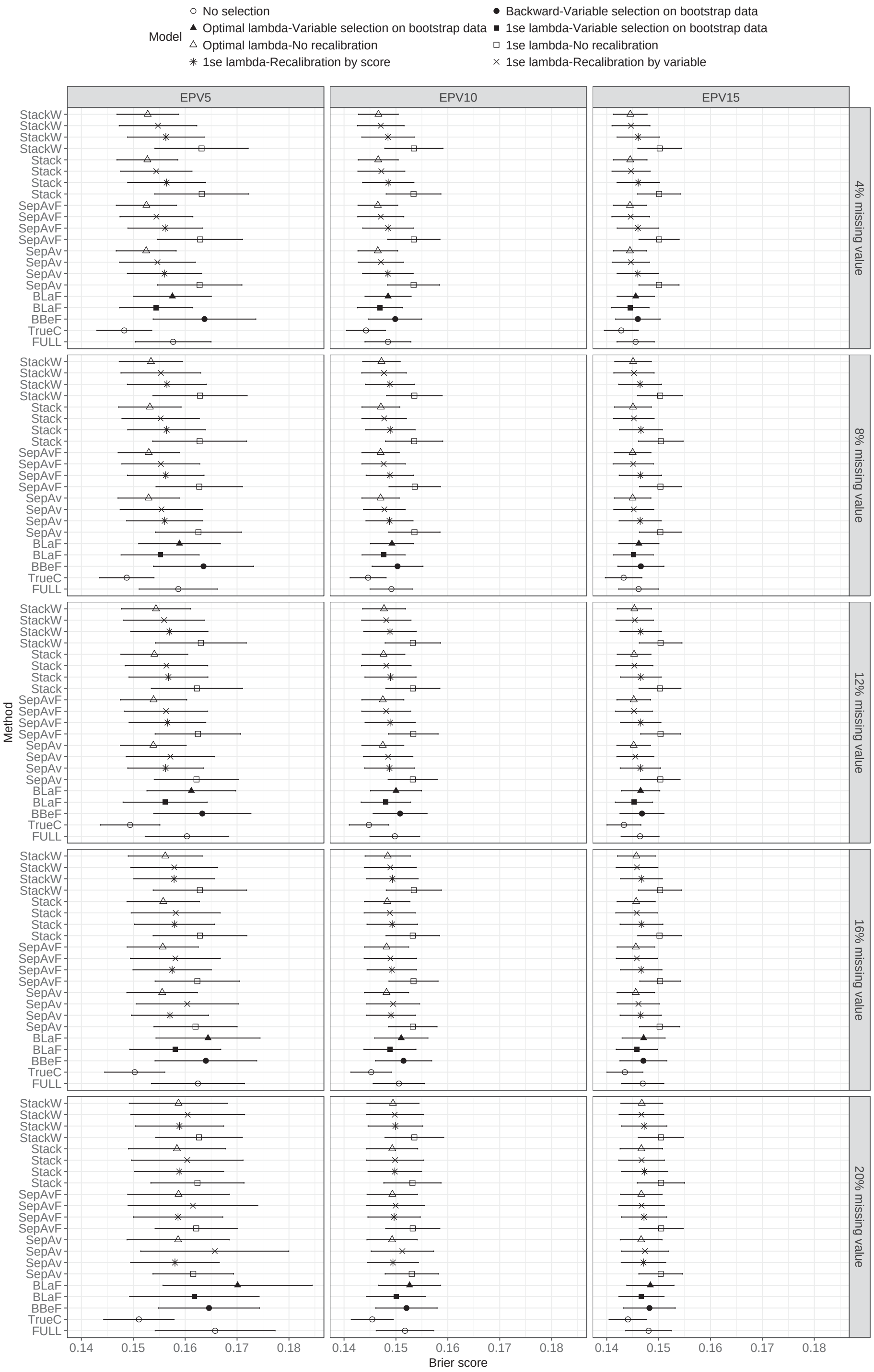
Model
No selection
Optimal lambda-Variable selection on bootstrap data
Optimal lambda-No recalibration
1se lambda-Recalibration by score
Backward-Variable selection on bootstrap data
1se lambda-Variable selection on bootstrap data
1se lambda-No recalibration
1se lambda-Recalibration by variable
EPV5
EPV10
EPV15
4% missing value
8% missing value
12% missing value
16% missing value
20% missing value
Method
StackW
Stack
SepAvF
SepAv
BLaF
BBeF
TrueC
FULL
0.14
0.15
0.16
0.17
0.18
Brier score

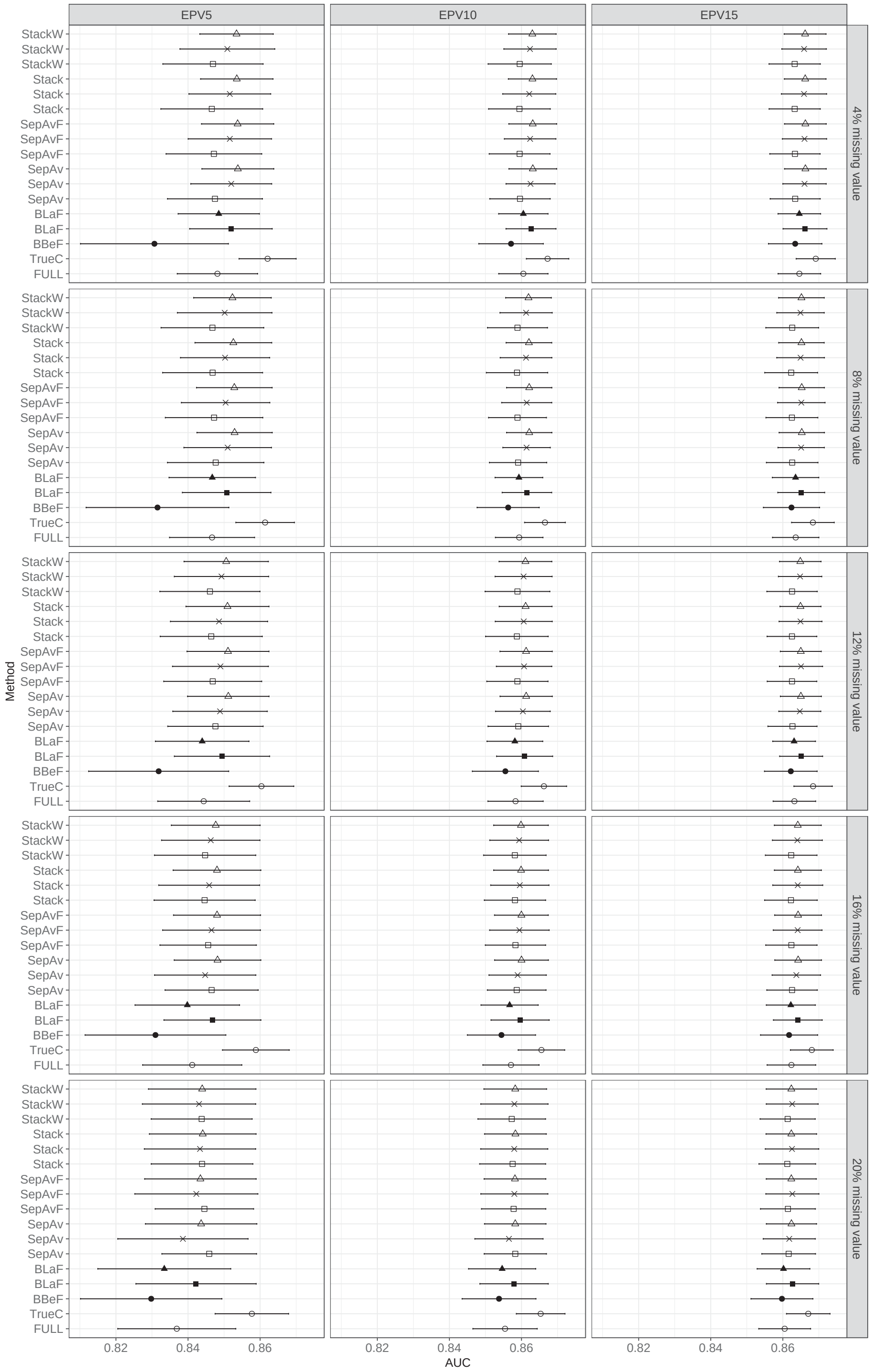

Figure S3: First data generating mechanism: Mean Brier score (top figure) and AUC (bottom figure) over 500 generated data sets for all scenarios with 10 imputed data sets. Black bars represent the empirical standard deviation

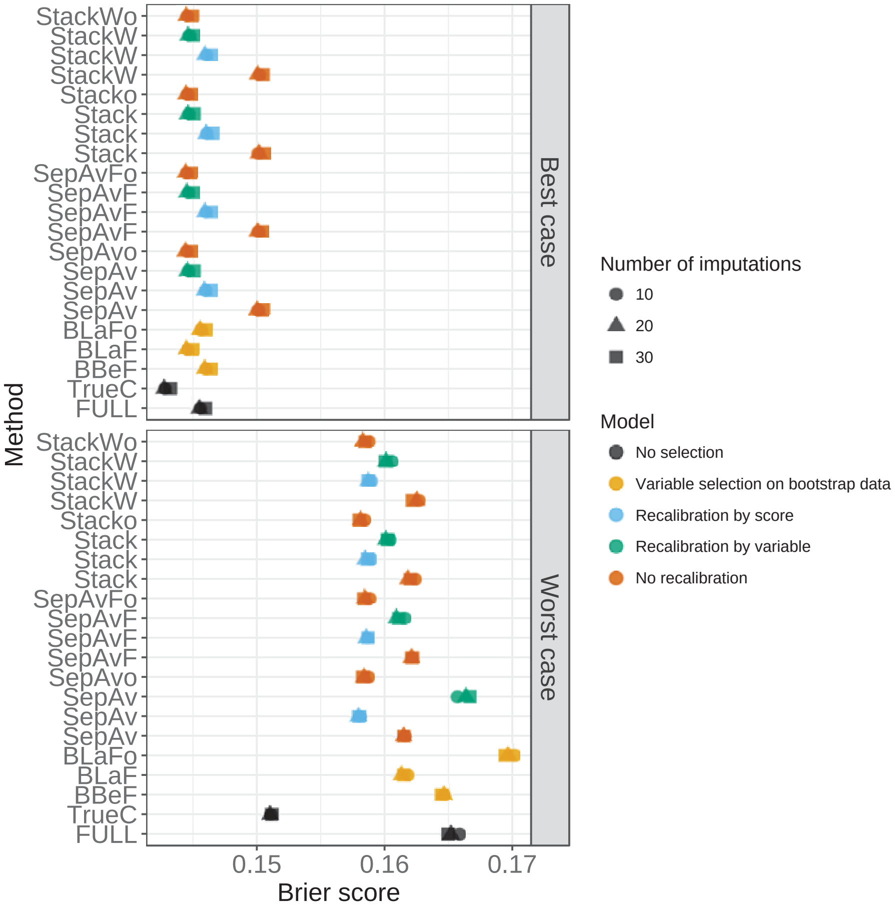
Method
StackWo
StackW
StackW
StackW
Stacko
Stack
Stack
Stack
SepAvFo
SepAvF
SepAvF
SepAvF
SepAvo
SepAv
SepAv
SepAv
BLaFo
BLaF
BBeF
TrueC
FULL
Best case
Worst case
0.15
0.16
0.17
Brier score
Number of imputations
10
20
30
Model
No selection
Variable selection on bootstrap data
Recalibration by score
Recalibration by variable
No recalibration

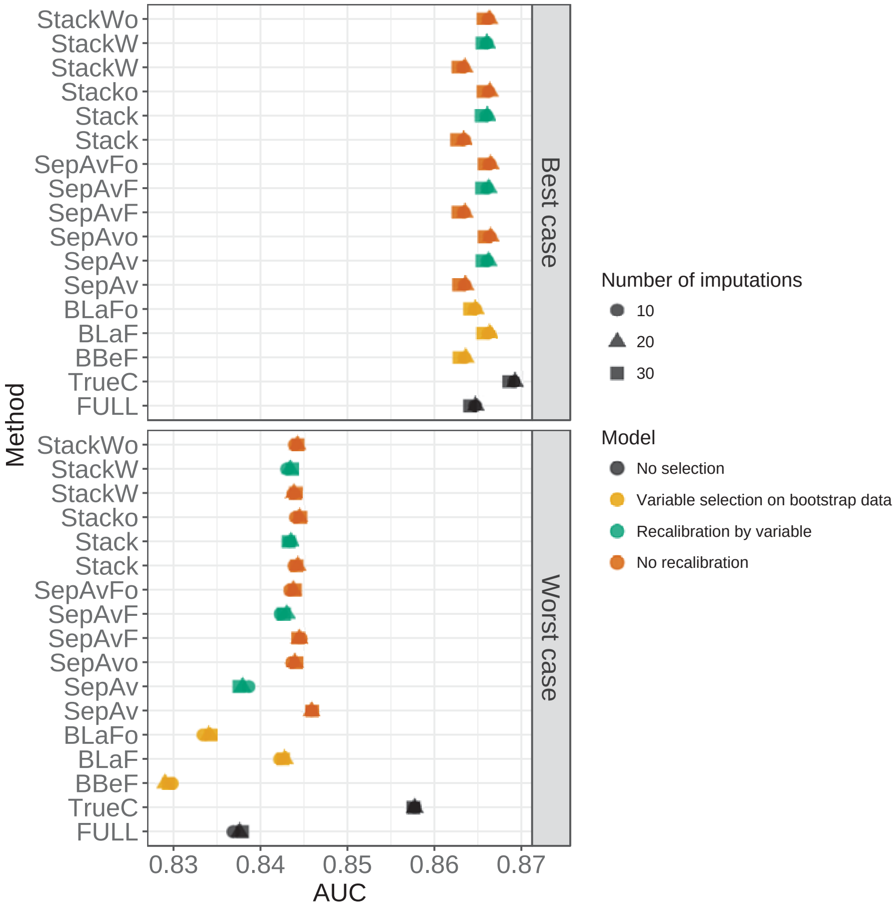

Figure S4: First data generating mechanism: The effect of the number of imputations on model performance in the two most extreme data scenarios. Brier score (top figure), AUC(bottom figure). Best case: 4% missing and 15 EPV; Worst case: 20% missing and 5 EPV

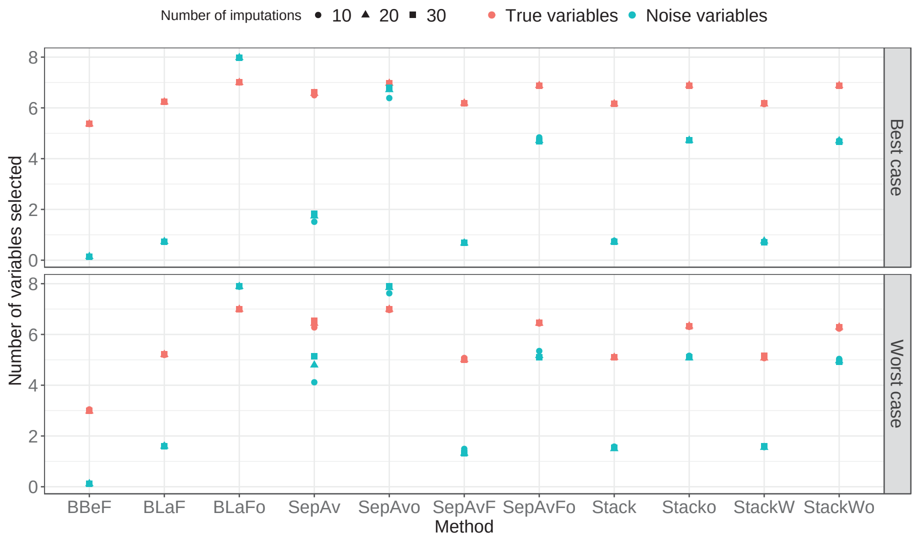

Figure S5: First data generating mechanism: The effect of the number of imputations on the number of selected variables in the two most extreme data scenarios. Best case: 4% missing and 15 EPV; Worst case: 20% missing and 5 EPV

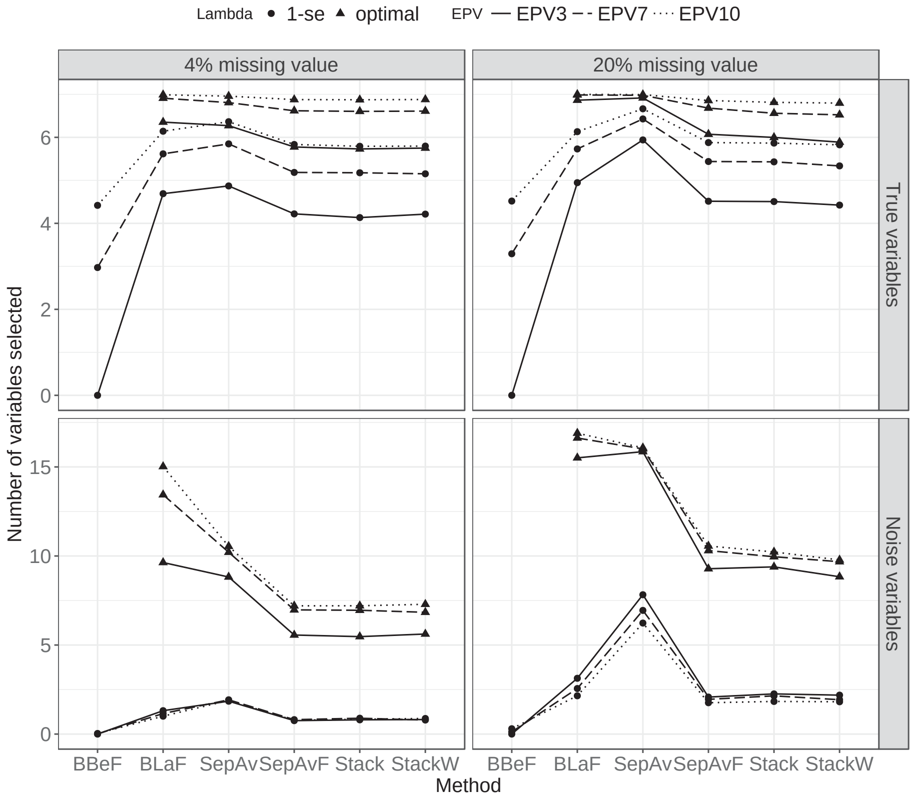

Figure S6: Second data generating mechanism: Number of selected variables for the two most extreme cases of missing values (4% and 20%) with 10 imputed data sets. The values for BBeF in data with EPV of 3 are not available due to convergence problems in many generated data sets.

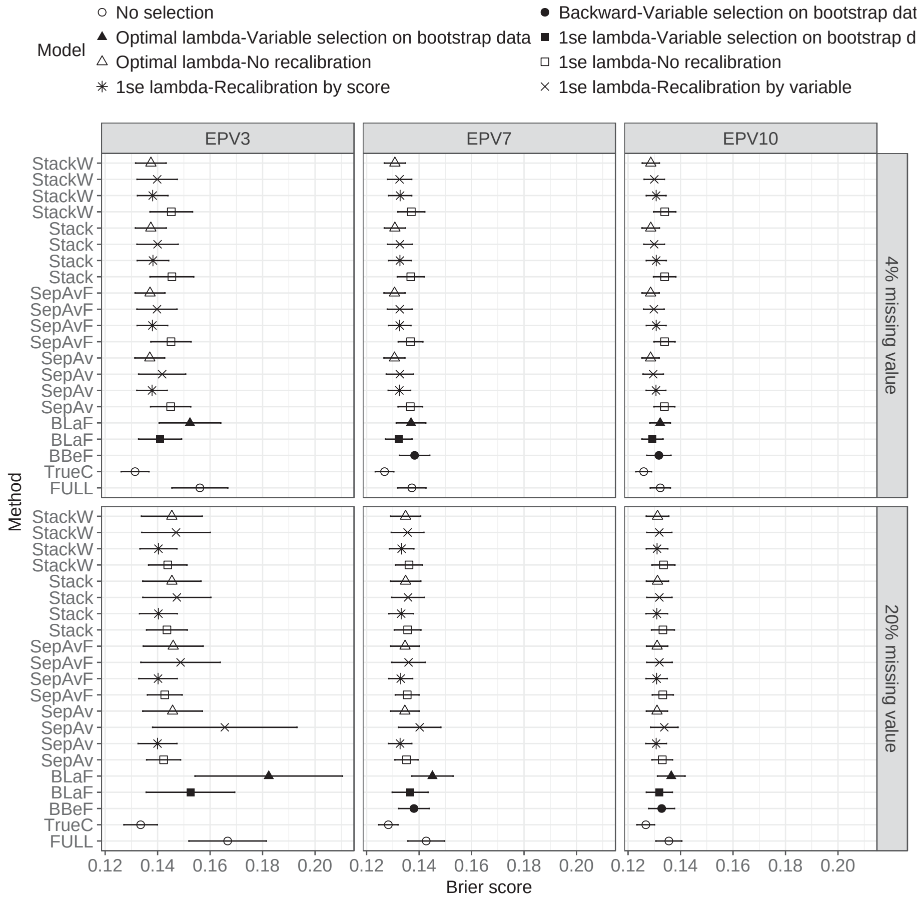
Model
No selection
Optimal lambda-Variable selection on bootstrap data
Optimal lambda-No recalibration
1se lambda-Recalibration by score
Backward-Variable selection on bootstrap dat
1se lambda-Variable selection on bootstrap d
1se lambda-No recalibration
1se lambda-Recalibration by variable
EPV3
EPV7
EPV10
4% missing value
20% missing value
Method
StackW
Stack
SepAvF
SepAv
BLaF
BBeF
TrueC
FULL
0.12 0.14 0.16 0.18 0.20
Brier score

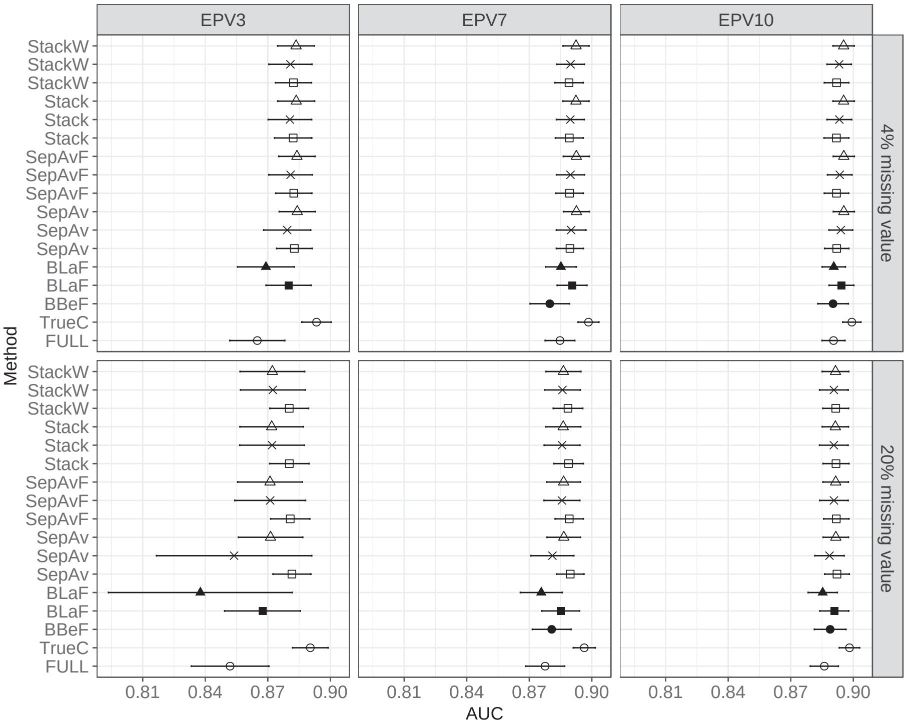

Figure S7: Second data generating mechanism: Mean Brier score (top figure) and AUC (bottom figure) over 500 generated data sets for the two most extreme cases of missing values (4% and 20%) with 10 imputed data sets. Black bars represent the empirical standard deviation.
